# Single Ingestion of Trehalose Enhances Prolonged Exercise Performance by Effective Use of Glucose and Lipid in Healthy Men

**DOI:** 10.3390/nu13051439

**Published:** 2021-04-24

**Authors:** Naomi Hamada, Tsuyoshi Wadazumi, Yoko Hirata, Mayumi Kuriyama, Kanji Watanabe, Hitoshi Watanabe, Nobuko Hongu, Norie Arai

**Affiliations:** 1 Department of Applied Food Science, Higashiosaka Junior College, 3-1-1, Nishidutsumigakuen-cho, Higashiosaka, Osaka 577-8567, Japan; 2 Faculty of Health and Well-Being, Kansai University, 1-11-1, Kaorigaoka-cho, Sakai-ku, Sakai, Osaka 590-8515, Japan; wadazumi@kansai-u.ac.jp (T.W.); yo-hirata@kwjc.kobe-wu.ac.jp (Y.H.); 3 Department of Food and Nutritional Science, Kobe Women’s Junior College, 4-7-2 Nakamachi, Minatojima, Chuo-ku, Kobe, Hyogo 650-0046, Japan; 4 Faculty of Nursing Sciences, Meiji University of Integrative medicine, Honoda Hiyoshi-cho, Nantan, Kyoto 629-0392, Japan; ma_kuriyama@meiji-u.ac.jp; 5 School of Health and Sports Sciences, Mukogawa Women’s University, 6-46 Ikebiraki, Nishinomiya, Hyogo 663-8558, Japan; kanji_w@mukogawa-u.ac.jp; 6 Research Center for Urban Health and Sports, Osaka City University, 3-3-138, Sugimoto, Sumiyoshi-ku, Osaka 558-8585, Japan; watanabe@sports.osaka-cu.ac.jp; 7 Department of Food and Human Life Science, Osaka City University, 3-3-138, Sugimoto, Sumiyoshi-ku, Osaka 558-8585, Japan; kay.hongu@gmail.com; 8 R&D Division, Hayashibara, Co., Ltd., 675-1, Fujisaki, Naka-ku, Okayama 702-8006, Japan; norie.arai@hb.nagase.co.jp

**Keywords:** trehalose, blood glucose, insulin, catecholamine, fat utilization, exercise performance

## Abstract

Trehalose increases blood glucose levels slowly and induces a slight insulin response. The present study aimed to study the effect of trehalose on prolonged exercise performance. The participants were 12 healthy men (age: 21.3 ± 0.9 y). After an overnight fast (12 h), they first exercised with a constant load (intensity: 40% $\dot{V}$O_2_peak) for 60 min using a bicycle ergometer. They continued to exercise with a constant load (40% $\dot{V}$O_2_peak) for 30 min between four sets of the 30-s Wingate test. After the 1st set, each participant ingested 500 mL water (control), 8% glucose, or 8% trehalose in three trials. These three trials were at least one week apart and were conducted in a double-blind and randomized crossover manner. Blood was collected for seven biochemical parameters at 12 time points during the experiment. The area under the curve of adrenaline after ingestion of trehalose was significantly lower than that for water and tended to be lower than that for glucose in the later stage of the exercise. Lower secretion of adrenaline after a single dose of 8% trehalose during prolonged exercise reflected the preservation of carbohydrates in the body in the later stage of the exercise. In conclusion, a single ingestion of trehalose helped to maintain prolonged exercise performance.

## 1. Introduction

During long-distance racing, such as marathon participation, muscle glycogen stored in skeletal muscle is continuously consumed and the depletion of glycogen causes a decline in performance in the later stage of the competition [1]. Ultra-high-intensity intermittent exercise (Wingate test) for 30-s reduces total muscle glycogen by approximately 20% [2]. The depletion of glycogen and associated hypoglycemia limit performance during prolonged exercise [3]. It is well-established that carbohydrate feeding before and during prolonged endurance exercise slows or reduces the onset of fatigue and increases endurance. Accordingly, the American College of Sports Medicine (ACSM) recommends that athletes consume 30–60 g of carbohydrate for every 1 h of exercise [4].

Numerous studies have investigated the effect of carbohydrate drinks on improving exercise performance by varying the type, amount, and method of ingestion [5,6,7,8,9,10,11]. Trehalose is a widely found natural substance that serves as a source of energy in most living organisms; it is found in several organisms, including bacteria, fungi, insects, plants, and invertebrates [12]. Trehalose, α-d-glucopyranosyl-α-d-glucopyranoside, is a disaccharide composed of two glucose molecules that are bound by an α-1, 1 glycosidic linkage. The linkage between the two glucose molecules contributes to its unique properties. Trehalose is not degraded in the mouth but is hydrolyzed into two glucose molecules by trehalase, a trehalose-specific digestive enzyme, in the brush border of the intestine and then absorbed [13]. When an oral trehalose tolerance test is performed, the rise in blood glucose levels is slower than that observed in an oral glucose tolerance test, and only a small amount of insulin is secreted [14,15,16]. Ingestion of trehalose before exercise suppresses the rise in blood glucose levels and insulin response and tends to show lower blood glucose levels than those after glucose ingestion [13].

This study examined the effect of single-dose trehalose on exercise performance and energy metabolism (i.e., combined prolonged constant-load exercise and Wingate test, see below, 2.3.1 Wadazumi Protocol) among healthy men. The respiratory exchange ratio (RER) remained significantly lower in the trehalose group than in the glucose group after carbohydrate feeding to the end of the protocol [17]. Other investigators have reported that trehalose ingestion induces only a slight insulin response in their experiments, suggesting that glucose metabolism is not enhanced by trehalose ingestion [14,16]. It has been hypothesized that a single dose of trehalose is more effective in improving exercise performance due to the preservation of carbohydrates during prolonged exercise. However, the physiological mechanisms by which this is achieved have not yet been elucidated. Therefore, this study aimed to clarify the mechanism by which a single dose of 8% trehalose assists in maintaining and improving performance in the later stage of exercise, in order to determine its usefulness in preventing or delaying the onset of fatigue in endurance athletes.

## 2. Materials and Method

### 2.1. Participants

Twelve healthy male college students with a daily exercise habit volunteered to participate in this study (Table 1). The objectives, methods, and risks of the study were fully explained to all participants, and written consent to participate in the study was obtained before participation. The participants were instructed to maintain their normal diet and physical activity during the experimental period. Strenuous physical activities and the intake of caffeine and alcohol were prohibited for 24 h before the experiment. Participants fasted from 21:00 on the day before the experiment to determine muscle glycogen depletion before participation.

### 2.2. Preliminary Testing

At least one week before the start of the experimental trials, the $\dot{V}$O_2_peak of each participant was determined using an electromagnetic brake-type bicycle ergometer (AEROBIKE 75XLIII, Konami Holdings Corporation, Tokyo, Japan) and an expired gas analyzer (AEROMONITOR AE-310S, Minato Medical Science Co., Ltd., Osaka, Japan). The exercise load (watts) at 40% $\dot{V}$O_2_peak was determined based on the detected values. Before the experiment, the participants practiced the Wingate test (POWERMAX-V III, Konami Holdings Corporation, Tokyo, Japan) and became familiar with it. The experiment was then conducted.

### 2.3. Experimental Design

The participants attended the laboratory at 9:00 for body composition measurements. Body weight measurements were taken to the nearest 0.5 kg in light training wear and without shoes using a balance beam scale (HBF-214, OMRON Corporation, Kyoto, Japan). Height was assessed to the nearest 0.1 cm using a wall-mounted stadiometer (UY-2, Uchida Yoko Co., Ltd., Tokyo, Japan). Percent body fat was evaluated using the HBF-214 (OMRON Corporation, Kyoto, Japan). Thereafter, the experiment was started in the laboratory at a room temperature of 25 °C and humidity of 50%. All participants completed three exercise trials with the same protocol and ingested either 8% glucose, 8% trehalose solution, or water (control). The three trials were at least 1 week apart and conducted in a double-blind and randomized crossover manner. Each trial consisted of approximately 5 h of combined cycling exercise, that is, constant-load cycling exercise at 40% $\dot{V}$O_2_peak and ultra-high intensity cycling intermittent exercise (Wingate test), according to the Wadazumi protocol.

#### 2.3.1. Wadazumi Protocol

**Figure 1:**
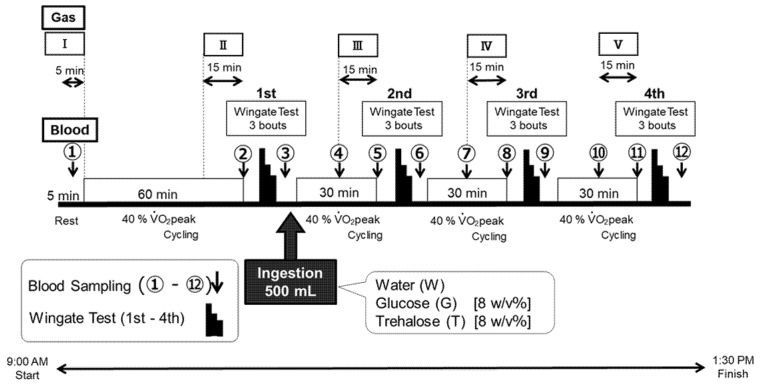
Experimental design. This cycling exercise protocol is a combination of constant-load exercise and the Wingate test. The intensity of the constant load was set at 40% $\dot{V}$O_2_peak for 60 min and 30 min × 3 times. A total of four sets of the Wingate test (30-s × 3 times) were performed. After the 1st Wingate test, the participants ingested a trial drink. Expired gas was collected at rest (I) and in the latter 15 min of the 60 min and 30 min constant-load exercise (II–V). The 12 points (①–⑫) indicated blood sample collection, RPE measurements, and Borg scale measurements.

**Figure 2:**
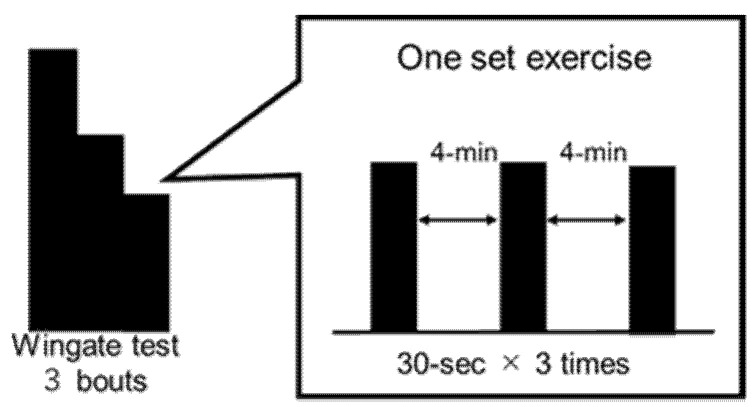
Wingate test.

This cycling exercise protocol is a combination of constant-load exercise and the Wingate test (Figure 1 and Figure 2) [17]. The participants performed 60 min of constant-load exercise using an electromagnetic brake-type bicycle ergometer at 40% $\dot{V}$O_2_peak intensity. After 60 min of constant-load exercise, the participants performed a total of four sets of the Wingate test combined with constant load exercise at 40% $\dot{V}$O_2_peak intensity for 30 min. After three (1st, 2nd, 3rd) Wingate tests, a constant-load exercise was performed for 30 min.

After completion of the Wingate test in the 1st set (1st), the participants ingested the test drink (see Section 2.4) and their performance over the subsequent three sets (2nd, 3rd, and 4th) of the Wingate test was observed. At the end of the 1st set of the Wingate test (1st), 500 mL water (trial W), 8 *w/v*% glucose solution (trial G), or 8 *w/v*% trehalose solution (trial T) was ingested within 5 min. The experimental design for ingestion of trial drinks by each participant is shown in Table 2. For fluid intake other than the single ingestion of the trial drink, the participants freely took water, and their intake was recorded. Expired gas was collected at rest (I) and in the latter 15 min of the 60 min and 30 min constant-load exercises (II–V).

#### 2.3.2. Wingate Test

The Wingate test using an electromagnetic brake-type bicycle ergometer was used as an indicator of exercise performance. The exercise mode is shown in Figure 2. A load of 0.075 kg for 1 kg body weight was applied to the pedal, and a 30-s Wingate test was performed three times with 4 min breaks between each test. Three Wingate tests were regarded as one set. The investigator instructed the participants to maintain their driving at full power in all sets and urged them to show their power by encouraging them.

### 2.4. Trial Drinks

Glucose (FUJIFILM Wako Pure Chemical Corporation, Osaka, Japan) and trehalose (Hayashibara Co., Ltd., Okayama, Japan) were used as carbohydrates in the experiments. Trehalose is less sweet than glucose and sucrose [12]; thus, it may be a more palatable drink for athletes. Glucose and trehalose were dissolved in water to a concentration of 8 *w/v*% to prepare a glucose solution (trial G) and a trehalose solution (trial T). The ACSM guidelines recommend carbohydrate ingestion of 30–60 g over 1 h of exercise [4]. The glucose (trial G) and trehalose (trial T) drinks consisted of approximately 40 g of carbohydrate dissolved in 500 mL water, as in our previous study [17]. Water (trial W) was used as a control.

### 2.5. Measurements

#### 2.5.1. Exercise Performance

In the Wingate test, “mean power value” for 30-s and “maximum power value” in which the power value per 5 s becomes the maximum value were detected. The “mean power value” was used as an index of exercise performance in this study.

#### 2.5.2. Blood Chemical Analysis

Seven items were measured in the blood chemical analysis: blood glucose, insulin, catecholamine 3 fractions (adrenaline, noradrenaline, and dopamine), free fatty acid (FFA), and lactic acid. Blood was collected from the median cubital vein by a physician or nurse at 12 points from the resting condition just before the experiment to the end of the experiment (Figure 1). While the participants were riding the bicycle, blood was collected at ④, ⑦, and ⑩ out of 12 points. At all other points, blood was collected when the participants dismounted and were in the sitting position. To collect blood intermittently, 14 mL of blood required for the experiment was collected by connecting an injection needle (18G 11/2, Terumo Corporation, Tokyo, Japan) to an extension tube and a three-way stopcock. The collected blood samples were processed for each measurement item, refrigerated or frozen, and analyzed by LSI Medience Corporation (Osaka, Japan).

#### 2.5.3. Expired Gas Analysis

To understand the use of energy substrates, expired gas was collected using an expired gas analyzer (Aero Monitor AE-310S, Minato Medical Science Co., Ltd., Osaka, Japan) (Figure 1I–V) to measure the RER, oxygen uptake ($\dot{V}$O_2_), and carbon dioxide production ($\dot{V}$CO_2_). Energy expenditure (EE) was calculated from $\dot{V}$O_2_ and CO_2_ values using the Weir formula, assuming that the percentage of protein was 12.5% [18]. Glucose oxidation (CHO) and fatty acid oxidation (FAO) were calculated using the following equations [19]:

$$\mathrm{EE}\;(\mathrm{kcal}/\min)=3.94\cdot \dot{V}O_{2}\;(L/\min)+1.11\cdot \dot{V}{\mathrm{CO}}_{2}\;(L/\min)$$

$$\mathrm{CHO}\;(g/\min)=4.344\cdot \dot{V}{\mathrm{CO}}_{2}\;(L/\min)-3.061\cdot \dot{V}O_{2}\;(L/\min)$$

$$\mathrm{FAO}\;(g/\min)=1.695\cdot \dot{V}O_{2}\;(L/\min)-1.701\cdot \dot{V}{\mathrm{CO}}_{2}\;(L/\min)$$

#### 2.5.4. Ratings of Perceived Exertion (RPE)

At the same time as blood collection, RPE were measured using the Borg scale [20].

### 2.6. Statistical Analysis

The results of this study were tested for normality using the Shapiro–Wilk test, and normality was confirmed. Thereafter, exercise performance was calculated with the first mean power value (watts) of the 1st set of Wingate tests as 100%, and the mean of three times per set was examined. For the blood chemistry analysis values, the area under the curve (AUC) was calculated for each item to evaluate changes in plasma after ingestion of the trial drink until the end of the experiment. Regarding the comparison between trial drinks based on each result, the test of sphericity of Mauchly was performed by one-way analysis of variance with repeated measure ANOVA. If the sphericity could not be presumed, the degrees of freedom were corrected using the Greenhouse–Geisser epsilon. Subsequently, the Bonferroni multiple comparison test was performed. Effect size (Cohen’s *d*) was calculated and determined as follows: small: 0.20 < *d* < 0.49, medium: 0.50 < *d* < 0.79, and large: 0.80 < *d*) [21]. SPSS Statistics ver. 25.0 (IBM Corporation) was used for statistical analysis. All measured values are expressed as the mean ± standard deviation (mean ± SD). The level of statistical significance was set at *p* < 0.05.

### 2.7. Ethics

This study was conducted in compliance with the Declaration of Helsinki on the basis of ethics, human rights, and the protection of personal information of participants. Ethical approval for this study was obtained from the ethics committee of the Kansai University Faculty of Health and Well-being (Approval No. 2018-17). The study was also registered at the University Hospital Medical Information Network Center (UMIN Center) (UMIN Study No.: UMIN000042752). All hard-copy (paper) study data were stored in a locked filing cabinet and electronic data on a secured network drive with access granted only to those working within the research lab.

## 3. Results

### 3.1. Mean Power Value

**Figure 3:**
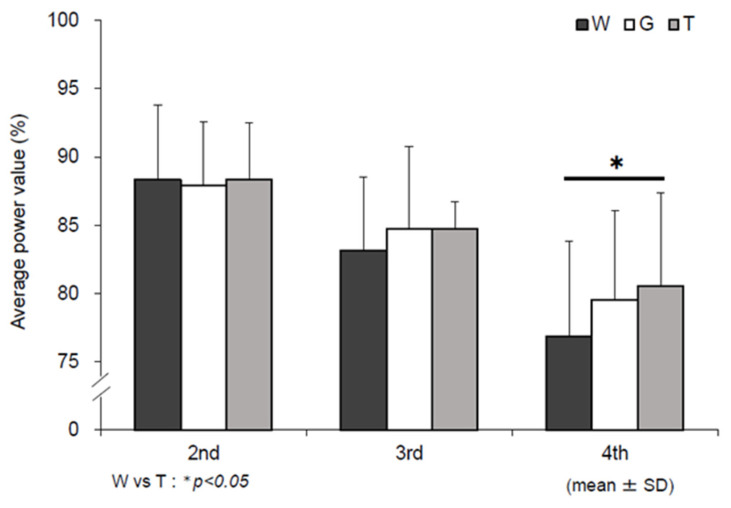
Average power assessments. W—water; G—glucose; T—trehalose. Comparison of mean power values in the Wingate test (2–4 sets) in each trial (water (W), glucose (G), trehalose (T)). The mean of three performances in each set (2nd, 3rd, and 4th) were compared when the first mean power value in the 1st set of the Wingate test (1st) was set at 100 in each trial. (*n* = 12); W vs. T: * *p* < 0.05.

The mean power values in the Wingate test in the three trials (W, G, and T) are shown in Figure 3. The mean power value was the highest in the 2nd set for all three trials and decreased in all trials as the set progressed. However, the decrease was smaller in trial T than in trials W and G, and a significant difference was observed in the main effect of the test-drink condition (*F* (2, 22) = 3.800, *p* = 0.038, $\eta_{p}^{2}$ = 0.257) in the 4th set, defined as the later stage of the exercise. In the comparison between the three trials, the value was significantly higher in trial T (80.5 ± 6.8%) than in trial W (76.9 ± 7.0%) (W vs. T, *p* = 0.017, *d* = 0.53). However, no significant difference was observed compared with trial G (79.5 ± 6.6%) (W vs. G, *p* = 0.387, *d* = 0.40).

### 3.2. Blood Glucose Level

**Figure 4:**
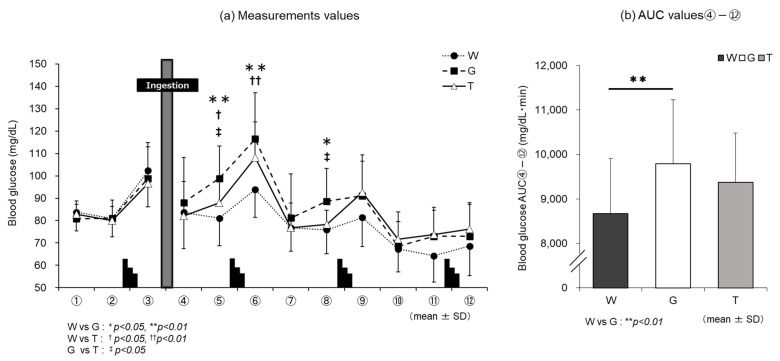
Changes in blood glucose levels for each trial. W—water; G—glucose; T—trehalose. Changes in blood glucose levels in each trial (water (W), glucose (G), trehalose (T)). Comparison between (**a**) measurements and (**b**) area under the curve (AUC) values ④–⑫ of each trial. (*n* = 12); W vs. G: * *p* < 0.05, ** *p* < 0.01; W vs. T: ^†^ *p* < 0.05, ^††^ *p* < 0.01; G vs. T: ^‡^ *p* < 0.05.

The changes in blood glucose levels in the three trials are shown in Figure 4a. In all three trials, blood glucose increased after the Wingate test for each set (③, ⑥, ⑨, ⑫). Fifteen minutes after the ingestion of each trial drink (④), there were no major differences in blood glucose levels among the three trials. However, at 30 min after ingestion (⑤), trial G (98.8 ± 14.4 mg/dL) showed a sharp increase, while trial T (87.9 ± 9.3 mg/dL) showed a mild increase and trial W (81.1 ± 12.3 mg/dL) showed a slight decrease. The three trials showed significant differences owing to the effect of carbohydrate drink consumption. The value in trial G was significantly higher than that in trial T (G vs. T, *p* = 0.040, *d* = 0.90; W vs. G, *p* = 0.001, *d* = 1.32; W vs. T, *p* = 0.038, *d* = 0.63). After the 2nd set of the Wingate test (⑥), there was no significant difference between trial T (108.1 ± 16.0 mg/dL) and trial G (116.5 ± 20.6 mg/dL), but trials T and G showed significantly higher values than trial W (93.9 ± 12.5 mg/dL) (T vs. G, *p* = 0.489, *d* = 0.46; W vs. T, *p* = 0.001, *d* = 0.99; W vs. G, *p* = 0.003, *d* = 1.33). After the 3rd set of the Wingate test (⑨–⑫), the values changed in almost the same manner in trials T and G.

Comparison of AUC ④–⑫ (Figure 4b) for each trial drink after ingestion until the end of the experiment revealed a significant main effect of the trial-drink condition (*F* (2, 22) = 7.325, *p* = 0.004, $\eta_{p}^{2}$ = 0.400). In the comparison of the three trials, the value was significantly higher in trial G (9796 ± 1435 mg/dL·min) than in trial W (8667 ± 1241 mg/dL·min) (G vs. W, *p* = 0.005, *d* = 0.84), and there was no significant difference between trial T (9377 ± 1102 mg/dL·min) and trial W.

### 3.3. Insulin Level

**Figure 5:**
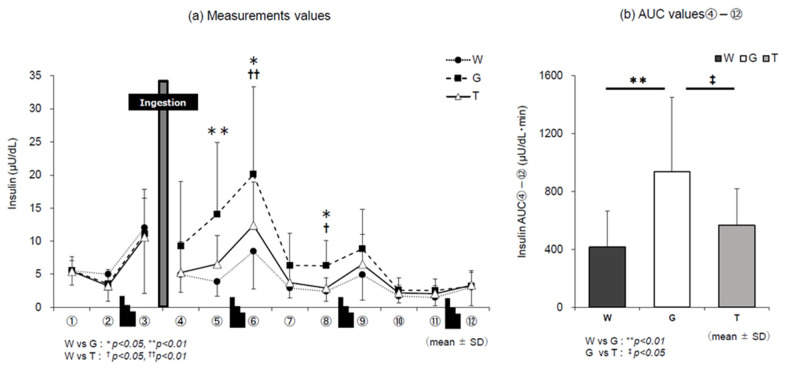
Changes in insulin levels for each trial. W—water; G—glucose; T—trehalose. Changes in insulin levels in each trial (water (W), glucose (G), trehalose (T)). Comparison between (**a**) measurements and (**b**) area under the curve (AUC) values ④–⑫ of each trial (*n* = 12). W vs. G: * *p* < 0.05, ** *p* < 0.01; W vs. T: ^†^ *p* < 0.05, ^††^ *p* < 0.01; G vs. T: ^‡^ *p* < 0.05.

The changes in insulin levels in the three trials are shown in Figure 5a. In all three trials, insulin levels changed according to changes in blood glucose levels. Comparison of the AUC values in points ④–⑫ (Figure 5b) after ingestion of the trial drink until the end of the experiment revealed significance in the main effect of the trial-drink condition (*F* (2, 22) = 13.563, *p* = 0.000, $\eta_{p}^{2}$ = 0.552). In the comparison between the three trials, the value was significantly lower in trial T (567 ± 254 μU/dL min) than in trial G (T vs. G, *p* = 0.028, *d* = 0.91).

### 3.4. FFA

**Figure 6:**
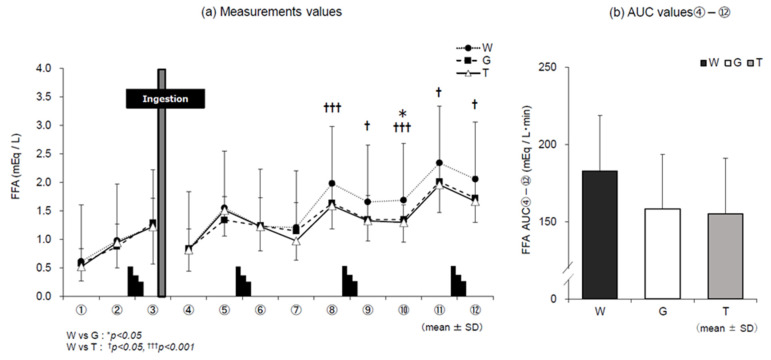
Changes in FFA levels for each trial. W—water; G—glucose; T—trehalose. Changes in free fatty acid level (FFA) in each trial (water (W), glucose (G), trehalose (T)). Comparison between (**a**) measurements and (**b**) area under the curve (AUC) values ④–⑫ of each trial (*n* = 12). W vs. G: * *p* < 0.05; W vs. T: ^†^ *p* < 0.05, ^†††^ *p* < 0.001.

Changes in FFA levels in the three trials are shown in Figure 6a,b. FFA levels were significantly lower in trial T than in trial W in the later stages of exercise (⑧–⑫). There were no statistical differences between the three trials in the main effect of AUC ④–⑫ after ingestion of the trial drink until the end of the experiment.

### 3.5. Catecholamines

AUC ④–⑫ of each catecholamine, and the results of the statistical analysis are shown in Table 3.

**Figure 7:**
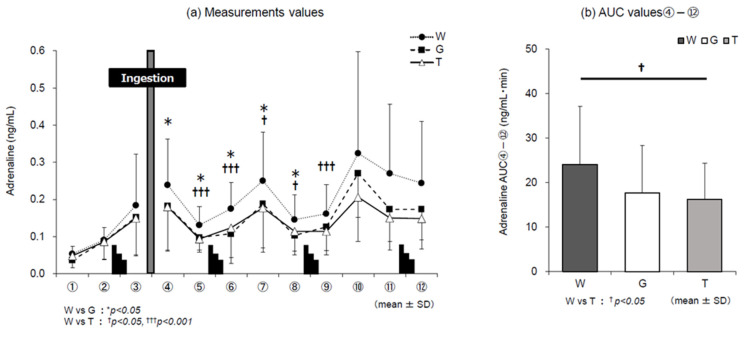
Changes in adrenaline levels for each trial. W—water; G—glucose; T—trehalose. Changes in adrenaline levels in each trial (water (W), glucose (G), trehalose (T)). Comparison between (**a**) measurements and (**b**) area under the curve (AUC) values ④–⑫ of each trial (*n* = 12); W vs. G: * *p* < 0.05; W vs. T: ^†^ *p* < 0.05, ^†††^ *p* < 0.001.

Changes in adrenaline levels (Figure 7a) were higher in trial W than in trials G and T until the end of the experiment. The values in trials T and G changed in almost the same manner until the middle of the exercise. However, a difference was observed after the end of the 3rd set of the Wingate test (⑩–⑫), and the values in trial T were lower than those in trial G. Comparison of AUC ④–⑫ (Figure 7b) for each trial drink after ingestion until the end of the experiment revealed significant differences in the main effect of the trial-drink condition (*F*(2, 22) = 6.793, *p* = 0.005, $\eta_{p}^{2}$ = 0.382). In the comparison between the three trials, the value in trial T was significantly lower than that in trial W (T vs. W, *p* = 0.010, *d* = 0.72).

**Figure 8:**
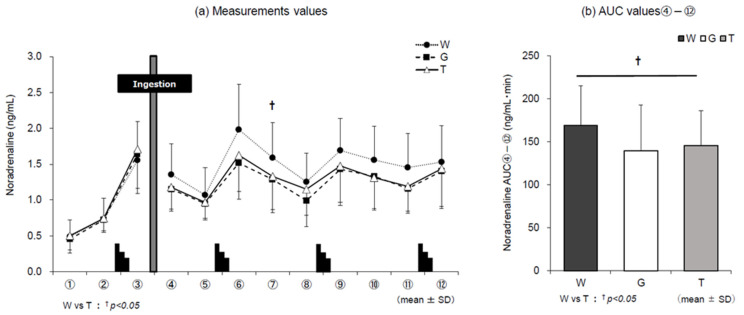
Changes in noradrenaline levels for each trial. W—water; G—glucose; T—trehalose. Changes in noradrenaline levels in each trial (water (W), glucose (G), trehalose (T)). Comparison between (**a**) measurements and (**b**) area under the curve (AUC) values ④–⑫ of each trial (*n* = 12); W vs. T: ^†^ *p* < 0.05.

Similar to adrenaline levels, the changes in noradrenaline levels (Figure 8a) were consistently higher in trial W than those in trials G and T after ingestion of the trial drinks until the end of the experiment. Comparison of AUC ④–⑫ (Figure 8b) for each trial drink after ingestion until the end of the experiment revealed significance in the main effect of the trial-drink condition (*F* (2, 22) = 4.453, *p* = 0.024, $\eta_{p}^{2}$ = 0.288). In the comparison between the three trials, the value in trial T was significantly lower than that in trial W (T vs. W, *p* = 0.017, *d* = 0.55). AUC ④–⑫ of dopamine was lower in trials G and T than in trial W after ingestion of the trial drink until the end of the experiment (*F* (2, 22) = 1.032, *p* = 0.373, $\eta_{p}^{2}$ = 0.086), although no significance was observed in the main effect.

### 3.6. RER, EE, CHO, and FAO

**Figure 9:**
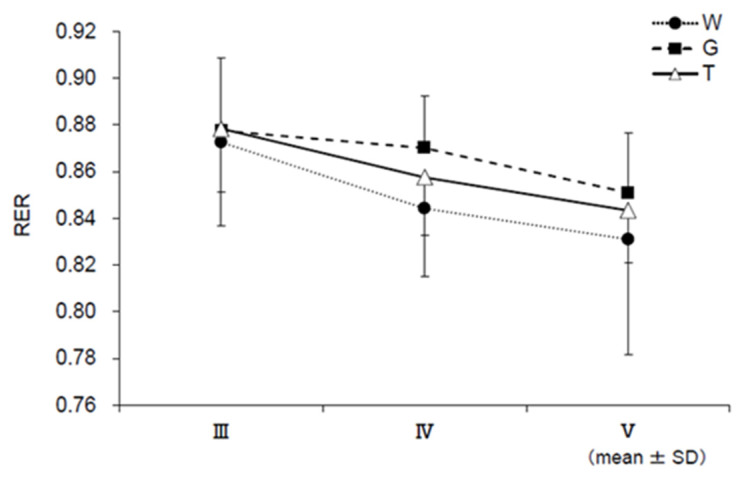
Respiratory exchange ratio (RER) assessments. W—water; G—glucose; T—trehalose. Comparison of RER for each trial (water (W), glucose (G), and trehalose (T)) (n = 12). Significance was found in the main effect only in IV (*p* < 0.05). In the Bonferroni multiple comparison test, there was no significant difference between the trials, but there was a significant trend in trial W vs. trial G (*p* = 0.080, *d* = 0.99), suggesting that the percentage of the use of carbohydrate was higher than that in trial T.

**Figure 10:**
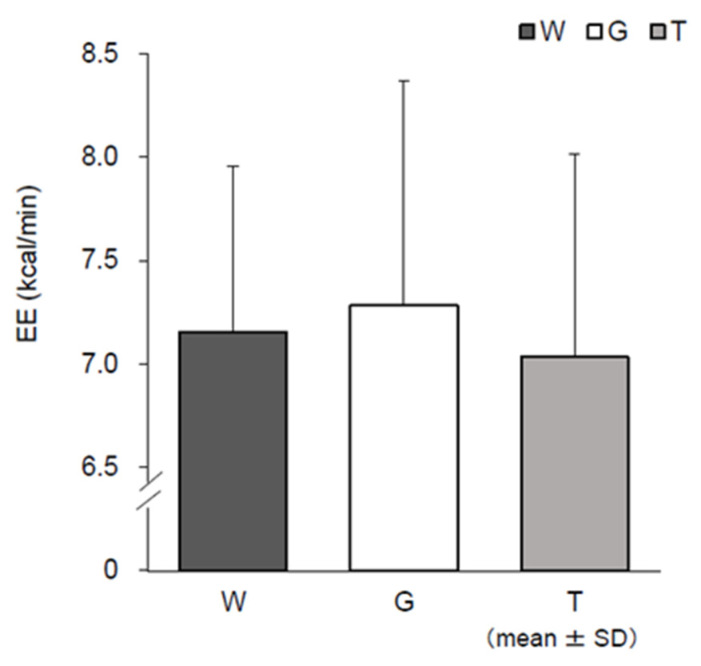
Energy expenditure (EE) assessments. W—water; G—glucose; T—trehalose. Comparison of mean EE (kcal/min) in each trial (water (W), glucose (G), trehalose (T)) in III–V (*n* = 12).

**Figure 11:**
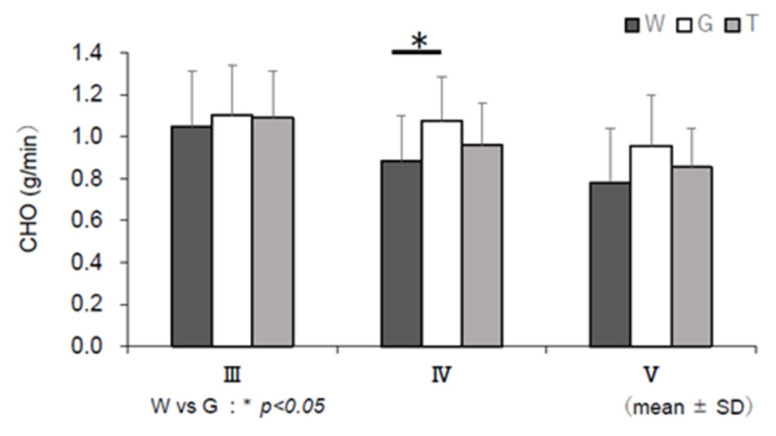
Glucose oxidation (CHO) assessments. W—water; G—glucose; T—trehalose. Comparison of CHO (g/min) in each trial (water (W), glucose (G), and trehalose (T)) in III, IV, and V (*n* = 12). W vs. G: * *p* < 0.05.

**Figure 12:**
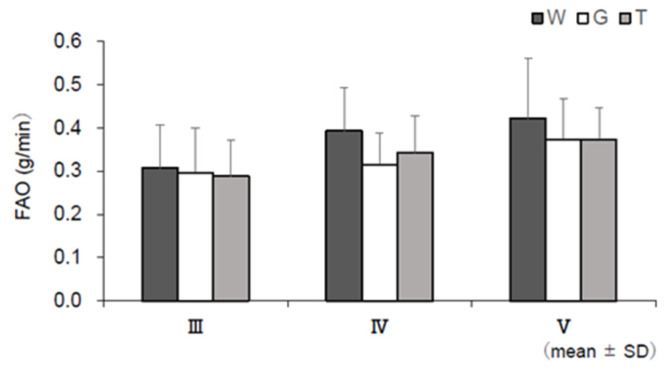
Fatty acid oxidation (FAO) assessments. W—water; G—glucose; T—trehalose. Comparison of FAO (g/min) in each trial (water (W), glucose (G) and trehalose (T)) in III, IV, and V (*n* = 12). W vs. G: * *p* < 0.05.

Figure 9 shows the changes in each RER during the last 15 min of constant-load exercise (III–V) based on the results of the expired gas analysis, excluding the resting condition. RER was lower in trial W in the later stages (IV and V) than in trials T and G. The main effect of IV (*F* (2, 22) = 4.008, *p* = 0.033, $\eta_{p}^{2}$ = 0.267) was significant. No significant difference was observed between trials T and G, but trial T showed lower values than trial G. For EE, the mean EE of III–V per minute was calculated (Figure 10). Although no statistical significance was observed, trial T showed lower values than trial G. Changes in CHO and FAO during the last 15 min of the constant-load exercise (III–V) are shown in Figure 11 and Figure 12. For CHO, the values in trial T were lower than those in trial G in IV and V, and significance was observed in the main effect of IV (*F*(2, 22) = 5.405, *p* = 0.012, $\eta_{p}^{2}$ = 0.329). In the comparison between the three trials, the value was significantly higher in trial G than in trial W (*p* = 0.040, *d* = 0.90). However, no significant difference was observed in the main effect of FAO, and no difference was observed between trials T and G in V.

### 3.7. Lactate, RPE, Amount of Free Water Intake

No difference was observed in lactate levels, RPE, or amount of free water intake between the three trials, and the changes were similar until the end of the experiment.

## 4. Discussion

In this study, we investigated the mechanism by which a single dose of trehalose was associated with the maintenance and improvement of performance in the later stage of the Wadazumi protocol, that is, combined constant-load exercise and the Wingate test, in healthy male college students with regular exercise habits. Our previous study showed that compared to a single dose of glucose, a single dose of trehalose did not lead to activation of glucose metabolism without reducing exercise efficiency, and exercise continued with a high lipid contribution rate [17]. We hypothesized that, compared with a single dose of glucose, a single dose of trehalose suppresses or slows glucose metabolism and continues with a high lipid contribution rate, thus preserving muscle glycogen. As a result, carbohydrates were preserved, and exercise performance was maintained and improved. In our Wingate test, one set of 30-s anaerobic exercise performed on a stationary bicycle comprised three bouts with a 4 min break between bouts (see Figure 2). A previous study reported that 70 to 80% of energy was supplied by glycolytic metabolism in a single set of Wingate test, resulting in a decrease in muscle glycogen of 20% [22]. In multiple bouts of the Wingate test, the performance of the 2nd and subsequent sets is considered to be affected by the uptake of blood glucose and muscle glycogen storage during a break period [23]. Parolin et al. (1999) reported that the decrease in the performance of the first and third bouts (total workload, watts) was caused by a marked decrease in muscle glycogen consumption due to glycolytic metabolism in the third and subsequent cycles, although the contribution of PCr degradation and aerobic metabolism remained unchanged [22]. Muscle glycogen was not quantified in this study. However, the participants in this study exercised more than the experimental protocol of Parolin et al. (1999), including the Wingate test and constant-load exercise [22]. There was no significant difference in performance in the later stages of exercise between trials T and G in this study. Therefore, it is presumed that muscle glycogen in both trehalose and glucose trials reduced to at least equivalent levels in the 2nd and subsequent sets. However, trehalose ingestion showed a smaller decline in performance than that in glucose ingestion, maintaining performance until the later stages of exercise. The potential mechanisms underlying this trend are discussed below.

### 4.1. Dynamics of Adrenaline

Catecholamines, hormones secreted from the adrenal medulla, include adrenaline, noradrenaline, and dopamine. The blood catecholamine level depends on the intensity of exercise. It rises sharply after an intensity of 50–70% $\dot{V}$O_2_peak [24,25,26]. A sharp increase has also been reported in intermittent exercise, in which high-intensity exercise is repeated for a shorter period of time with maximum power [27,28]. It has also been reported that blood glucose levels decrease and catecholamine levels increase, reflecting the depletion of carbohydrates in the body [24,29,30,31]. Adrenaline is considered to react most markedly out of the three catecholamines [32]. In this study, adrenaline levels increased after high-intensity exercise and in the later stages of exercise (Figure 7a). The results of trial W were consistently higher than those of trials T and G from the beginning to the end of the experiment. This was probably because the depletion level of carbohydrates in the body increased in the later stage of exercise due to the absence of carbohydrate intake during exercise. In trial T, adrenaline AUC ④–⑫ after ingestion of the trial drink was significantly lower than that in trial W (Figure 7b) and tended to be lower than that in trial G in the later stage of the exercise (Figure 7a, ⑩–⑫). These results suggested that among the three trials, the depletion of carbohydrate in the body was the slowest in trial T, and carbohydrate was preserved. For the preservation of carbohydrates, it is effective to use lipids efficiently or save carbohydrate use as an energy source.

### 4.2. Use of Lipids

Concerning the use of lipids, triglycerides stored in adipose tissues and skeletal muscle are degraded and FFA are released into the bloodstream during exercise at 40% $\dot{V}$O_2_peak or less, or when the level of carbohydrate depletion in the body increases. At these points, lipids are preferentially used as an energy source. The results of this study suggest that FFA in trial W remained high until the end of the experiment because the depletion of carbohydrates in the body progressed without carbohydrate ingestion and the use of lipids as an energy source increased. However, it was considered that trials T and G showed lower values than trial W because of carbohydrate ingestion (Figure 6a,b).

### 4.3. Use of Carbohydrate

Blood glucose levels sharply increased in trial G after the ingestion of glucose, while the increase in blood glucose level was slower in trial T than in trial G (Figure 4a). Trehalose, a disaccharide consisting of two bound glucose molecules, is absorbed after being hydrolyzed into glucose by trehalase, a trehalose-specific degrading enzyme secreted in the small intestine [16]. Compared to trial G, in which glucose was absorbed, it was presumed that blood glucose levels did not sharply increase in trial T, as trehalose needed time to be degraded into glucose by trehalase. It has been reported in a study in which a glucose tolerance experiment was conducted with suppression of insulin secretion that an increase in the blood glucose level itself enhances glucose metabolism regardless of insulin [33,34]. A higher RER ($\dot{V}$CO_2_/$\dot{V}$O_2_) results in a higher contribution of carbohydrates as the energy substrate and a lower RER results in a higher contribution of lipids [35]. According to the results of RER (Figure 9), it was considered that glucose levels increased due to the intake of carbohydrates in trials T and G, showing higher values than trial W. There was no difference immediately after ingestion of the trial drink (III) between trials T and G. Trial G showed high values until the later stages of exercise (IV, V); thus, glucose metabolism continued to increase. However, it was presumed that trial T did not rely on glucose metabolism or the glycolytic system to the same extent as trial G. EE (Figure 10) and CHO (Figure 11) were lower in trial T than in trial G. Accordingly, it was presumed that trial T was able to perform the same amount of work (exercise efficiency) with less EE and continued exercise.

### 4.4. Trend of Insulin

Incretin is a gastrointestinal hormone that is secreted into the blood from intestinal endocrine cells after meals and stimulates pancreatic β cells to promote insulin secretion [29]. There are two types of incretins: glucose-dependent insulinotropic peptide (GIP) and glucagon-like peptide-1 (GLP-1). Both induce insulin secretion before glucose reaches pancreatic β cells [36,37]. Yoshizane et al. reported that the secretion of GIP after ingestion of trehalose under resting conditions was markedly lower than that after glucose ingestion [16]. GIP is secreted from K cells in the upper small intestine by the stimulation of nutrients such as carbohydrates and lipids. Trehalose hardly stimulates K cells in the process of absorption in the gastrointestinal tract [37]. These results suggest that insulin AUC ④–⑫ in trial T (Figure 5b) was significantly lower than that in trial G (*p* = 0.028, *d* = 0.91). Typically, when blood glucose levels are increased by meals and insulin is secreted from the pancreas and reaches the muscle, GLUT4 moves from inside the cell to the cell membrane surface and increases glucose uptake into skeletal muscle [38]. The incorporated glucose is immediately used, or glycogen is synthesized and stored if not used immediately. Insulin activates glucokinase and phosphofructokinase (PFK), which are glycolytic rate-limiting enzymes, and hexokinase in skeletal muscle and increases glycolytic activity. These results indicate that trial G increased insulin secretion and glucose metabolism, while trial T decreased insulin secretion, resulting in decreased glucose metabolism and enhanced preservation of carbohydrates than those in trial G (Figure 5a,b).

Accordingly, adrenaline secretion was lower at the time of trehalose ingestion in the later stage of the exercise, suggesting preservation of carbohydrates in the body. As blood glucose slowly increased, which is characteristic of trehalose, and insulin secretion was mild, trial T did not enhance the glycolytic system as much as trial G but suppressed the use of carbohydrates. This supports the findings of a previous study by Wadazumi et al. [17] that ingestion of a trehalose drink is effective in maintaining and improving performance until the later stage of exercise.

In trial G, it was shown that a single dose of glucose led to increased glucose metabolism until the later stage of the exercise, increased EE, and decreased exercise efficiency. In trial T, however, it was presumed that carbohydrate waste was suppressed without reducing the exercise efficiency and exercise continued with a high lipid contribution rate than that in trial G. Consequently, carbohydrate was preserved and exercise performance was maintained and improved.

### 4.5. Applications and Future Research 

Glucose ingestion led to increased glucose metabolism until the later stage of exercise, increased energy expenditure, and decreased exercise efficiency. However, the ability to suppress the rapid rise in blood glucose level and insulin after trehalose ingestion did not reduce exercise efficiency and led to the preservation of glucose necessary for maintaining performance. In addition, the fact that performance could be maintained until the end of exercise even with a single ingestion suggests that trehalose can reduce time lost to ingestion during a race, as well as reduce gastrointestinal problems due to repeated glucose supplementation. The findings of this study suggest that trehalose could be a useful ingredient in carbohydrate drinks that aim to improve athletic performance during prolonged exercise such as marathons and other endurance events. Specific studies to address this possibility are required in future research.

## 5. Conclusions

A single ingestion of an 8% trehalose drink resulted in a gradual increase in blood glucose levels and a significantly lower insulin level than did a single ingestion of an 8% glucose drink, resulting in more efficient use of glucose without causing a rapid decrease in blood glucose level. It is speculated that this results in the preservation of carbohydrates in the body, maintaining and improving performance until the later stage of exercise.

## Figures and Tables

**Table 1:** Characteristics of the participants included in the study.

*n* = 12	Age	Height	Weight	%Fat	$\dot{V}$O_2_peak	Load (Peak)	40%$\dot{V}$O_2_peak	Load (40%$\dot{V}$O_2_peak)
	(years)	(cm)	(kg)	(%)	(mL/min)	(watts)	(mL/min)	(watts)
Mean	21.3	176.3	70.0	17.9	3193.4	278.0	1277.4	102.9
SD	0.9	5.6	8.5	1.9	649.5	38.0	259.8	16.1

**Table 2:** Experimental design—participant numbers and randomized crossover trials.

Participant No.	Trial 1	Trial 2	Trial 3
1	T	G	W
2	T	W	G
3	G	T	W
4	W	G	T
5	G	W	T
6	W	T	G
7	T	W	G
8	G	W	T
9	W	G	T
10	G	T	W
11	W	T	G
12	T	G	W

W: water, G: glucose, T: trehalose.

**Table 3:** Statistical results for catecholamine AUC ④–⑫ (*n* = 12).

**a: One-Way Analysis of Variance with Repeated Measures ANOVA**					***p*-Value**	$\eta_{p}^{2}$
	**W**	**G**		**T**		
Adrenaline	24.1 ± 13.1	17.7 ± 10.6		16.2 ± 8.1	0.005 **	0.382
Noradrenaline	169.4 ± 45.6	139.8 ± 52.9		145.6 ± 40.9	0.024 *	0.288
Dopamine	4.9 ± 1.4	4.3 ± 1.4		4.4 ± 1.2	0.373	0.086
**b: Bonferroni Multiple Comparison Test between Trial Drinks**						
	**W vs. G**		**W vs. T**		**G vs. T**	
	*p*-value	Cohen’s *d*	*p*-value	Cohen’s *d*	*p*-value	Cohen’s *d*
Adrenaline	0.112	0.54	0.010 *	0.72	1.000	0.16
Noradrenaline	0.089	0.60	0.017 *	0.55	1.000	0.12
Dopamine	0.574	0.41	0.847	0.35	1.000	0.09

* *p* < 0.05, ** *p* < 0.01 (mean ± SD) W—water; G—glucose; T—trehalose. (**a**) One-way analysis of variance with repeated measurements; (**b**) Bonferroni multiple comparison test between trial drinks; * *p* < 0.05, ** *p* < 0.01.

## Data Availability

The data presented in this study are available on request from the corresponding author and the permission of all parties involved in the study.
